# Relationship between body positivity and body neutrality with body image, self-esteem, mindfulness and gratitude

**DOI:** 10.1038/s41598-025-22423-2

**Published:** 2025-11-05

**Authors:** Anna Lipińska

**Affiliations:** Department of Clinical Psychology and Psychoprophylaxis, Institute of Psychology, University of Szczecin, Szczecin, Poland

**Keywords:** Psychology, Health care

## Abstract

Despite the growing popularity of body positivity and body neutrality on social media, their scientific differentiation remains unclear. Body positivity involves acceptance and a positive attitude toward one’s body regardless of societal ideals, whereas body neutrality emphasizes a non-judgmental focus on body functionality. This study aimed to examine the relationships between body positivity and body neutrality, self-esteem, body image, mindfulness, and gratitude. A total of 201 adults (56.7% women; *M* = 28.11; *SD* = 11.84) completed the Rosenberg Self-Esteem Scale, Body Evaluation Scale, Five Facet Mindfulness Questionnaire: Short-Form, Gratitude Questionnaire and a survey measuring the self-reported level of body positivity and body neutrality. Body positivity and body neutrality were positively correlated with all psychological variables (*p* < 0.05). Body positivity was predicted by self-esteem and body image (*R²* = 0.41), whereas body neutrality was predicted by self-esteem, gratitude, and mindfulness (*R²* = 0.30). The high and low groups differed significantly in all variables except for the Observing facet of mindfulness. The value of the shared variance coefficient between body positivity and neutrality (*ρ²* = 0.23) indicated the presence of distinct constructs. The findings support the distinctiveness of body positivity and body neutrality, and highlight their relevance to psychological well-being.

## Introduction

The beginnings of the *body positivity movement* are associated with the organisation founded by Connie Sobczak and Elizabeth Scott in 1996, The Body Positive^1^. However, the activities of activists promoting the normalisation of diverse images, especially of those who are socially excluded, began even earlier. The idea grew out of the activism of citizens of the USA in the 1960 s as an expression of resistance to the growing social discourse that stigmatised bodies deviating from the canon, in particular the promoted body thinness^2^. The term body positivity as well as the related body neutrality^3^ and the associated activities have become widespread in the online space over the past decade, especially through the social media platform Instagram^4^. In 2019, the hashtag #bodypositive on Instagram contained a collection of 6.1 million posts^5^, and within a year, this number had increased to nearly 12.6 million posts^6^. Despite its growing popularity in mass culture, body positivity is still a relatively new topic in scientific research^6^. In the mainstream media, body positivity is defined as an attitude of rejecting unattainable standards of beauty and the messages that support them, and respecting the diversity of people’s body shapes and sizes^5^. Some of the more mainstream conceptions of body positivity focus on respecting people of all body shapes and sizes, resisting societal beauty ideals and portraying self-love^2,7^.

The impact of social media content tagged as body positive on the responders’ wellbeing is not unambiguous. Some research findings suggest that being exposed to body positive content was associated with greater body satisfaction, lower negative affect and greater positive affect^6^. Other researchers point out increased positive mood with body satisfaction and negative mood remaining unaffected^8^. Other studies propose that being exposed to body positive social media content can be associated with heightened self-objectification^5,9^. In terms of body neutrality in social media, women who consumed body neutrality social media content, in comparison to consuming thin-ideal content, reported significantly higher state body image scores, less upward appearance comparisons and more positive thoughts about their appearance^10^. However, a thematic analysis of the TikTok content posted under body positivity and body neutrality hashtags highlighted three themes apparent across both types of content (resisting societal ideologies with subtheme: normalizing insecurities, (re)producing disordered content with subtheme: toxic (body) positivity promotes the need for neutrality, and social critique)^11^.

For the purposes of the present study, the definition of body positivity was adopted as the acceptance of one’s body and the feeling of positive affect towards one’s body image or the pursuit of it^12^. It was important for the planned analyses to distinguish between body positivity and body neutrality. Body neutrality was previously defined as a non-judgemental approach to one’s own body. Meaning minimizing the importance of appearance and instead shifting the attention towards body functionality, on what the body allows them to do^11,13^. Other authors conclude that body neutrality contains many elements of positive body image, such as body appreciation, body functionality appreciation and body image flexibility while promoting a neutral approach. Thus, it’s not a new concept in the field of psychology^14^. The present study used the definition of body neutrality referring to an attitude of acceptance and lack of judgement of one’s body, focusing on the possibilities it provides^15^.

In the present study, self-esteem is defined as a relatively constant and conscious positive or negative attitude towards the self^16^. Previously, a relationship between self-esteem and satisfaction with one’s body image has been noted^17^. According to Thomas Cash, body image is described as including all thoughts, beliefs, feelings and behaviours regarding one’s own physical appearance^18^. Self-esteem also has a connection to mindfulness and experiencing positive affect and psychological well-being^19^.

Mindfulness is broadly defined as a type of unforced, non-judgemental, present-centred awareness. Every thought, feeling or sensation that enters the field of attention is noted and accepted as it is^20^. For the purposes of the study, a model was adopted which assumes that mindfulness consists of five aspects of functioning: *activation with awareness*, *non-reactivity*, *describing*, *observing* and *not judging*^21^. Not judging, i.e. observing thoughts, emotions and sensory impressions without evaluating them, seems to reflect part of the attitude of body neutrality^22^. Prior research supports using mindfulness-based interventions to increase positive body image and reduce body dissatisfaction^23,24^. What’s more, practicing mindfulness can reduce the tendency of body checking and avoiding situations that were once associated as strong triggers for negative body judgement, thoughts or behaviors^25^.

Previous research^26,27^ supports the assumption that there is a link between mindfulness and gratitude. Gratitude has been defined as a general tendency to recognise and respond with feelings of gratitude to the kindness of others and the pleasant experiences that they can provide^28^. Research into the relationship between gratitude and self-esteem does not provide clear conclusions that would allow this relationship to be assessed^29,30^. To the best of the researcher’s knowledge, there are currently no psychological studies on the direct relationships between body positivity and body neutrality with self-esteem, mindfulness and gratitude.

In summary, the body positivity social movement aligns more with the idea of respecting, embracing and loving one’s body regardless of sociocultural body and appearance standards, while the body neutrality movement is more associated with recognizing and valuing the body for what it can do^31^. Other research suggests a stage model proposing that people may transition through stances of internalization of appearance standards towards those of body appreciation, liberation, and then neutrality^32^, which may imply that body positivity is a stage in transition towards body neutrality. Similarly, Darwin and Miller defined body neutrality as a form of radical body positivity that emerged as a reaction to the critiques of the body positivity movement^33^. The present study tests the supposed distinction, or lack of, between body positivity and body neutrality. The aim of the study is to explore the relationship between body positivity and body neutrality with the variables of body image, self-esteem, mindfulness and gratitude. The results obtained may contribute to clarifying the existing definitions of body positivity and body neutrality in social discourse and to broadening the available knowledge about them.

The following hypotheses were tested in the study:

### Hypothesis 1:

Body positivity and body neutrality are significantly related to self-esteem, body image, mindfulness and gratitude.

### Hypothesis 2:

There is a difference in the level of self-esteem, body image, mindfulness and gratitude between people with high body positivity and those with low body positivity.

### Hypothesis 3:

There is a difference in the level of self-esteem, body image, mindfulness and gratitude between people with high body neutrality and those with low body neutrality.

## Methods

### Participants

A total of 201 people aged between 18 and 70 took part in the study (*M* = 28.11; *SD* = 11.84). The respondents included 114 women (56.72%) and 87 men (43.28%). The majority of the respondents had secondary education (*n* = 119; 59.20%) and were residents of large cities with a population of 101,000–500,000 (*n* = 82; 40.80%). The sample was divided into subgroups of people with low (*n* = 158; 78.61%) and high (*n* = 43; 21.39%) body positivity, and low (*n* = 141; 70.15%) and high (*n* = 60; 29.85%) body neutrality. In addition, four subgroups were distinguished among the study participants, respectively, with: low body positivity and body neutrality (*n* = 126; 62.69%), low body positivity and high body neutrality (*n* = 32; 15.92%), high body positivity and low body neutrality (*n* = 15; 7.46%), and high body positivity and body neutrality (*n* = 28; 13.93%).

### Statistical analyses

The calculations were performed using *IBM SPSS Statistics 26.0*. A 95% confidence interval (*p* < 0.05) was adopted to reject the null hypothesis. Due to the measurement scale of the selected variables, correlation analyses were performed using Spearman’s *rho* coefficient to estimate the level of co-occurrence of the selected variables. In order to determine the differences in the average level of variables between two groups, a Student’s t-test for independent samples was performed, and between four groups, an analysis of variance in the intergroup plan with a post hoc test. In addition, a multiple regression analysis was performed with a stepwise method of selecting individual predictors of the body positivity and body neutrality. A priori estimates indicate that the obtained sample size (*N* = 201) was sufficient to detect medium statistical effects in correlational analyses (*r* = 0.30, required *N* = 84), Student’s t tests (*g* = 0.50, required *N* = 128), and regression analyses (*f*² = 0.15, required *N* = 77)^34,35^. The database did not contain any missing values or influential outliers.

### Procedure

The study was carried out using an online questionnaire created in Google Forms. The participants were informed in writing about the scope of the collected data and the purpose of the study. Each person interviewed gave written consent to participate in the study. The inclusion criteria were the age of majority and communicative knowledge of the Polish language. The exclusion criteria were being a minor and not knowing the Polish language. The Body Evaluation Scale (BES) distinguishes between different subscales for women and men and therefore excludes responses from people who declare a different gender identity. The subjects were recruited by posting a survey on the social networking site Facebook. The snowball method was used to increase the recruitment of subjects. The research project was approved by the Bioethics Committee for Research Projects of the Institute of Psychology at the University of Szczecin (No. KB 58/2024) and the study was conducted consistently with the Declaration of Helsinki. All participants gave their free and informed consent.

## Measures

Demographic data such as age, gender identity, education and place of residence of the respondents were collected by means of a self-report survey designed by the researcher. Due to the lack of a research tool measuring the variables of body positivity and body neutrality adapted to Polish conditions, self-description survey was used. To ensure a high enough level of accuracy in the selection of survey items, the evaluation was carried out by a group of competent judges composed of psychology researchers associated with the Institute of Psychology of the University of Szczecin. The decision was made to select the items ‘Do you accept your body and strive to like it?’ to reflect body positivity and ‘Do you accept your body for what it allows you to experience in life, regardless of whether you find it attractive?’ to reflect body neutrality. Study participants were assigned to subgroups to test the hypotheses based on content analysis of survey responses. Responses of Definitely not, Rather not and Neither yes nor no classified the respondents into a group with low level of body positivity or body neutrality. Meanwhile, responses of Rather yes or Definitely yes classified into a group with high level of body positivity or body neutrality.

The Rosenberg Self-Esteem Scale (SES)^16^ in its Polish adaptation^36^ allows for the measurement of the general level of self-esteem, interpreted as a relatively unchanging, conscious, positive or negative attitude of the subject towards his or her own self. The scale consists of 10 diagnostic statements. The recipient declares their agreement with the statements presented on a four-point scale, from 1 – I strongly agree to 4 – I strongly disagree. The tool demonstrates high internal consistency (α = 0.81–0.83) and stability measured using the test-retest method (*r* = 0.50–0.83). Additionally, the Polish adaptation of the SES showed relatively high validity, which was confirmed through factor analysis^36^. In the study, Cronbach’s alpha is a = 0.89 with an accepted confidence interval of 95%.

The Body Evaluation Scale (BES)^37^ adapted to Polish conditions^38^ enables the measurement of the respondents’ attitude towards their body. It consists of 35 test items organised into three subscales, different for men and women. The subscales for men are: Physical Attractiveness, Body Strength, Physical Condition. The subscales for women are: Sexual Attractiveness, Weight Control, Physical Condition. The respondent answers the statements on a five-point Likert scale from 1 – I have strong negative feelings to 5 – I have strong positive feelings, where 3 expresses a neutral attitude towards a given aspect of physicality. In the present study, Cronbach’s alpha is a = 0.95 in the test version for men and a = 0.96 in the test version for women with an accepted confidence interval of 95%.

The Five Facet Mindfulness Questionnaire: Short-Form (FFMQ-SF)^39^ in the adaptation to Polish conditions^40^ is used to measure mindfulness, which is recognised in five facets: Observing, Describing, Acting with Awareness, Nonjudging, Nonreactivity. The test consists of 24 items. The measurement is made by determining the extent to which the subjects agree with the statements presented on a five-point Likert scale from 1 – (Almost) never to 5 – (Almost) always. The Polish version of the FFMQ-SF^40^ achieved high reliability measured by Cronbach’s alpha coefficient, ranging from 0.73 to 0.86 (except for the Nonreactivity subscale: 0.65–0.66). Furthermore, the five-factor structure of the questionnaire was confirmed, and convergent validity was demonstrated through correlations with neuroticism and rumination. Cronbach’s alpha is equal to a = 0.81 with an accepted confidence interval of 95%.

The gratitude questionnaire (GQ-6)^28^ adapted to Polish conditions^41^ is used to measure the level of gratitude in the respondent, understood as the tendency to perceive pleasant experiences and the kindness of other people. The scale consists of 6 items. The measurement is carried out by asking the respondent to evaluate the extent to which he or she agrees with the statements on a seven-point Likert scale ranging from 1 – Strongly disagree to 7 – Strongly agree. The GQ-6 demonstrates good reliability (α = 0.71) and satisfactory psychometric properties for a one-factor structure (*GFI* = 0.96; *AGFI* = 0.91; *RMSEA* = 0.09), as well as high external validity based on correlations with Big Five personality traits and satisfaction with life^41^. The Cronbach’s alpha coefficient is a = 0.79 with an assumed confidence interval of 95%.

## Results

Firstly, demographic data characterising each specified subgroup has been established. Table 1 shows the detailed differentiation of the study group in terms of the body positivity and body neutrality variables according to the values of the answers given.

**Table 1:** Response frequencies for demographic variables in the specified subgroups.

Variable	Body positivity		Body neutrality	
	Low (*n* = 158)	High (*n* = 43)	Low (*n* = 141)	High (*n* = 60)
	*n* (%)	*n* (%)	*n* (%)	*n* (%)
Gender				
Female	96 (60.76)	18 (41.86)	82 (58.16)	32 (53.33)
Male	62 (39.24)	25 (58.14)	59 (41.84)	28 (46.67)
Education				
Elementary school	3 (1.90)	1 (2.33)	4 (2.84)	0 (0)
Middle school	3 (1.90)	0 (0)	3 (2.13)	0 (0)
Vocational school	1 (0.63)	1 (2.33)	1 (0.71)	1 (1.67)
High school	96 (60.76)	23 (53.49)	87 (61.70)	32 (53.33)
University	55 (34.81)	18 (41.86)	46 (32.62)	27 (45.00)
Living area zone				
Rural	28 (17.72)	5 (11.63)	21 (14.89)	12 (20.00)
Urban (< 50 000 citizens)	21 (13.29)	3 (6.98)	20 (14.18)	4 (6.67)
Urban (50 000–100 000 citizens)	20 (12.66)	7 (16,28)	20 (14,18)	7 (11,67)
Urban (101 000–500 000 citizens)	64 (40.51)	18 (41.86)	56 (39.72)	26 (43.33)
Urban (> 500 000 citizens)	25 (15.82)	10 (23.26)	24 (17.02)	11 (18.33)

### Psychological correlates of body positivity and body neutrality

The results of the correlation analyses (Table 2) indicate that both body positivity and body neutrality correlated positively and statistically significantly (*p* < 0.001) with individual psychological variables. The highest value of the Spearman’s rho coefficient was obtained for self-esteem and body positivity (*ρ* = 0.61; *p* < 0.001; 95%*CI*[0.45;0.64]) as well as self-esteem and body neutrality (*ρ* = 0.50; *p* < 0.001; 95%*CI*[0.38; 0.59]). The Observing component of mindfulness correlated the least with both variables and co-occurred with the intensity of body positivity (*ρ* = 0.15; *p* < 0.05; 95%*CI* (0.01;0.28) and body neutrality (*ρ* = 0.17; *p* < 0.05; 95%*CI*[0.04;0.31]).

**Table 2:** Results of the correlation analysis using spearman’s Rho coefficients.

Variable	Body positivity			Body neutrality		
	ρ	95%CI		ρ	95%CI	
		LL	UL		LL	UL
Body image	0.55***	0.45	0.64	0.39***	0.26	0.50
Self-esteem	0.61***	0.52	0.69	0.50***	0.38	0.59
Gratitude	0.38***	0.25	0.49	0.39***	0.27	0.50
Mindfulness	0.46***	0.34	0.56	0.45***	0.33	0.56
Nonreactivity	0.31***	0.18	0.43	0.33***	0.20	0.45
Observing	0.15*	0.01	0.28	0.17*	0.04	0.31
Acting with Awareness	0.38***	0.26	0.50	0.31***	0.18	0.43
Describing	0.31***	0.18	0.43	0.30***	0.16	0.42
Nonjudging	0.34***	0.21	0.46	0.33***	0.20	0.45

Note. 95%*CI* = 95% confidence interval; LL = lower limits; UL = upper limits. * *p* < 0.05; *** *p* < 0.001.

In addition, the analyses showed a positive and statistically significant (*ρ* = 0.49; *p* < 0.001; 95%*CI*[0.38;0.59]) correlation between body neutrality and body positivity. In addition, the value of the shared variance coefficient obtained (*ρ*² = 0.23; *p* < 0.001) indicates that 22.9% of the variance of the body neutrality variable is explained by the body positivity variable and vice versa. This correlation is moderately strong, while indicating that these variables cannot be considered identical (*ρ*² < 0.70).

Subsequent multiple regression analyses showed that the significant predictors of body positivity included self-esteem (β = 0.43; *p* < 0.001) and body image (β = 0.31; *p* < 0.001). Self-esteem (β = 0.31; *p* < 0.001), gratitude (β = 0.16; *p* < 0.05) and mindfulness (β = 0.18; *p* < 0.05) for body neutrality. Both models were well-fitted to the data (*p* < 0.001) and explained 41% (*R*^*2*^ = 0.41) and 30% (*R*^*2*^ = 0.30) of the variance of these variables, respectively. Table 3 shows detailed results of the multiple regression analyses. The results support the assumption that the higher the level of self-esteem and body image, the higher the body positivity, and the higher the level of self-esteem, gratitude and mindfulness, the higher the level of body neutrality.

**Table 3:** Results of multiple regression analyses.

Predictor	*R* ^2^	F	B	SE	β	95%CI	
						LL	UL
Body positivity							
Self-esteem	0.41	69.90***	0.08	0.01	0.43***	0.06	0.10
Body image			0.02	0.01	0.31***	0.01	0.03
Body neutrality							
Self-esteem	0.30	28.40***	0.06	0.02	0.31***	0.03	0.10
Gratitude			0.03	0.01	0.16*	0.01	0.05
Mindfulness			0.02	0.01	0.18*	0.01	0.03

Note. B = unstandardized beta coefficient; β = standardized beta coefficient; 95%CI = 95% confidence interval; LL = lower limits; UL = upper limits. * *p* < 0.05; *** *p* < 0.001.

### Differences in the intensity of psychological variables between groups

The results of the Student’s t-test for independent samples (Table 4) indicate that both the group of people with high body positivity and the group of people with high body neutrality had significantly (*p* < 0.01) higher average scores on most psychological variables. The exception was the observing facet, the intensity of which did not differ (*t*(199) = 1.55; *p* > 0.05) between people with low (*M* = 16.61; *SD* = 3.02) and high (*M* = 15.44; *SD* = 3.48) body positivity. Furthermore, the obtained values of the Hedges’ *g* coefficients indicate moderate (< 0.50) and high (< 0.80) strengths of the identified effects^42^. For both groups, the strongest differences were shown for the *self-esteem* variable.

**Table 4:** Results of student’s t-test for independent samples.

Variable	Body positivity				t	g
	Low (*n* = 158)		High (*n* = 43)			
	M	SD	M	SD		
Body image	27.44	15.32	45.16	13.84	6.86***	1.18
Self-esteem	27.18	5.11	33.84	4.09	7.88***	1.35
Gratitude	29.83	6.53	34.30	5.92	4.06***	0.70
Mindfulness	76.11	11.09	86.61	12.68	5.33***	0.91
Nonreactivity	13.98	3.82	16.30	4.21	3.46***	0.59
Observing	14.61	3.02	15.44	3.48	1.55	0.27
Acting with Awareness	15.94	3.66	18.54	3.76	4.10***	0.70
Describing	16.58	3.69	18.63	3.67	3.24**	0.56
Nonjudging	15.01	3.71	17.70	4.55	4.00***	0.69

Variable	Body neutrality				t	g
	Low (*n* = 141)		High (*n* = 60)			
	M	SD	M	SD		
Body image	27.65	16.13	39.63	14.88	4.93***	0.76
Self-esteem	26.89	5.17	32.65	4.43	7.54***	1.16
Gratitude	29.52	6.54	33.77	5.95	4.33***	0.66
Mindfulness	75.31	10.64	85.53	12.71	5.88***	0.90
Nonreactivity	13.77	3.81	16.13	3.99	3.96***	0.61
Observing	14.41	3.05	15.67	3.18	2.64**	0.41
Acting with Awareness	15.80	3.59	18.12	3.90	4.08***	0.63
Describing	16.47	3.71	18.30	3.62	3.22**	0.50
Nonjudging	14.85	3.71	17.32	4.31	4.10***	0.63

Note. M = mean; SD = standard deviation; g = Hedges’ g. ** *p* < 0,01; *** *p* < 0,001.

Based on the results of the analysis of variance in the intergroup plan (Table 5), differences were shown in the average level of self-esteem (*F*(3.197) = 32.69; *p* < 0.001; η^2^ = 0.33), body image (*F*(3.197) = 19.06; *p* < 0.001; η^2^ = 0.23), mindfulness (*F*(3.197) = 16.26; *p* < 0.001; η^2^ = 0.20) and gratitude (*F*(3.197) = 10.23; *p* < 0.001; η^2^ = 0.14) between the compared groups. The obtained values indicate high strengths of the identified effects (η^2^ > 0.14).

**Table 5:** Results of the analysis of variance in the intergroup plan.

Group	Coefficient	Body image	Self-esteem	Gratitude	Mindfulness
Low body positivity – low body neutrality (*n* = 126)	M	25.73	26.26	28.92	74.34
	SD	14.96	4.93	6.51	10.30
Low body positivity – high body neutrality (*n* = 32)	M	34.19	30.81	33.41	83.09
	SD	15.08	4.13	5.33	11.50
High body positivity – low body neutrality (*n* = 15)	M	43.87	32.13	34.53	83.40
	SD	16.95	4.14	4.44	10.32
High body positivity – high body neutrality (*n* = 28)	M	45.86	34.75	34.18	88.32
	SD	12.15	3.84	6.66	13.64
	F	19.06***	32.69***	10.23***	16.26***
	η^2^	0.23	0.33	0.14	0.20

*Note*. M = mean; SD = standard deviation. *** *p* < 0.001.

Furthermore, the Bonferroni post hoc tests indicate that there are no significant differences (*p* > 0.05) in the strength of all variables between the group of people with low body positivity and high body neutrality and the group of people with high body positivity and low body neutrality. Likewise, between the group of people with high body positivity and low body neutrality and people with high body positivity and body neutrality. In addition, no significant differences were found in the average level of mindfulness (*t*(58) = 1.84; *p* > 0.05) and gratitude (*t*(58) = 0.48; *p* > 0.05) between people with low body positivity and high body neutrality and the group of people with high body positivity and body neutrality.

## Discussion

The aim of the present study was to determine whether body positivity and body neutrality have a significant relationship with self-esteem, body image, mindfulness and gratitude. The results provide evidence of a positive and statistically significant correlation between body positivity and body neutrality and the chosen variables (Hypothesis 1). The multiple regression analyses that were conducted showed that self-esteem and body image are significant predictors of body positivity. On the other hand, self-esteem, gratitude and mindfulness are significant predictors of body neutrality. The obtained results support the assumption that self-esteem and body image correlate positively with body positivity. Self-esteem, gratitude and mindfulness correlate positively with body neutrality. The results obtained are consistent with the current understanding of body positivity, which includes a positive body image^12^, and also indicates a positive relationship with self-esteem. The relationship between self-esteem and body image satisfaction is confirmed by previous research^17^.

Observed differences should be interpreted with adopted definitions and tools being taken into consideration. Currently BES is the only body image measuring tool adapted to be used on the Polish population, however its design forces responders to judge individual parts of their body and parameters such as its strength^38^. In the present study body positivity was adopted as the acceptance of one’s body and the feeling of positive affect towards one’s body image or the pursuit of it^12^. Whereas body neutrality includes non-judging attitude towards one’s body^15^, which possibly could have been reflected in the neutral assessment of their body image by the respondents, resulting in no positive or negative relationship between body image and body neutrality. The results achieved may differ if other aspects of body image were assessed, such as body appreciation, body functionality appreciation or body image flexibility, which have been previously associated with body neutrality^15^.

Mindfulness as an important predictor of body neutrality can be distinguished by the aspect of *not judging*, understood as a nonjudgemental observation of thoughts, emotions and sensory impressions, in this case regarding one’s body^22^. The adapted definition of body neutrality emphasized focusing on the possibilities the body provides and didn’t include judging its appearance. Such an approach could possibly be more strongly reflected in gratitude rather than positive or negative body image evaluation measured by BES. Previous research shows that gratitude has a strong direct effect on body appreciation^43^, which may be tied to body neutrality as previously suggested^15^. To the best researcher’s knowledge, there are currently no other psychological studies on relationships between body positivity and body neutrality with self-esteem, mindfulness and gratitude that would allow a comparison of the results.

The analyses conducted revealed a positive and statistically significant correlation between body neutrality and body positivity. At the same time, the value of the common variance coefficient indicates that these variables cannot be considered identical. Therefore, it is recommended to explore the analysis of these variables separately in future studies on attitudes towards body image. When comparing body positivity and body neutrality, body image, gratitude and mindfulness predictors may be crucial in their differentiation.

The existence of differences in the level of chosen variables depending on whether the person is characterised by high body positivity (Hypothesis 2) and high or low body neutrality (Hypothesis 3) is confirmed by the results of the analysis of the Student’s t-test for independent samples. The group of subjects with high body positivity, as compared to the group with low body positivity, as well as the group with high body neutrality, as compared to the group with low body neutrality, had significantly higher average scores for most of the variables examined. Only the facet of mindfulness – *observing* – did not differ significantly between people with low and high body positivity. Subsequently, the intergroup research was deepened by distinguishing four groups of subjects characterised by high or low levels of body positivity and body neutrality. The results of the analysis of variance show differences between the groups in the average level of self-esteem, body image, mindfulness and gratitude. The obtained results of differences in the analysed groups support the distinctiveness of body positivity and body neutrality. Some researchers argue against the distinction of body neutrality by interpreting it as an attitude that includes some elements of a positive body image, such as flexibility and appreciation of its functionality^14^. This may indicate an inconsistency in the definitions of body neutrality and positive body image used by researchers and encourage further research to unify the understanding of these phenomena.

The present study fulfilled its purpose of characterising the relationship between body positivity and body neutrality with the variables of body image, self-esteem, mindfulness and gratitude, proving their positive interdependence and providing new information on the functioning of body positive and body neutral people. The obtained results can be used in the future to test the impact of consuming body-positive and body-neutral content on specific aspects of the recipients’ functioning.

### Study limitations

In assessing the reliability of the present study, demographic limitations and the resulting slight variation in the age and education of the study group should be taken into account. The characteristics of the respondents did not take into account any potential physical disabilities, which can play a significant role in the perception and evaluation of one’s body – both its attractiveness and condition – and shape their experience differently from that of non-disabled people^44^.

Due to the cross-sectional nature of the study, it is not possible to conclude cause-and-effect relationships between the analyzed variables. The obtained results should be interpreted only as co-occurrences, which can be the basis for further longitudinal or experimental studies, allowing to determine the direction of the observed relationships.

Another limitation of the study is the restriction of the respondents’ gender identity to the binary division of gender due to the design of the BES tool. In the future, it is recommended to conduct research extended to the context of minority groups.

The limitations of the study include the necessity to use a self-description survey of the author’s own design to measure variables resulting from the lack of standardised tools to measure body positivity and body neutrality. The definitions of body positivity and body neutrality used for their measurements may have not been specific enough, which could contribute to the findings. The study used an original measurement of body positivity and body neutrality based on single items, which may limit the validity and reliability of the measurement of these constructs. The number of used categories might have an effect on data variety and should be considered in future studies. Although the questions were verified by competent judges and their content was based on current theoretical definitions, the use of multi-item instruments could provide a more complex and precise capture of the analyzed attitudes. Additionally, the use of single-point tools could have affected the precision of the measurement, thus increasing the risk of type II error. Future studies could benefit from using more multifaceted definitions and measurements, for example by including subscales of not only physical attractiveness but also body appreciation, body functionality appreciation and body image flexibility as it has been linked with both body positivity and body neutrality^15^. It is recommended to repeat the study using a standardised psychometric tool adapted to Polish conditions. Alternatively, qualitative research could provide a more in-depth and detailed understanding of body positivity and body neutrality. However, it is essential to acknowledge the potential for researchers’ subjectivity and the restricted generalizability of the findings^45^ while using this method in future studies.

## Data Availability

The data that support the findings of this study are openly available at OSF and can be accessed at: https://osf.io/jeyb7/.
